# Delayed Gadolinium-Enhanced MRI of Cartilage (dGEMRIC) Shows No Change in Cartilage Structural Composition after Viscosupplementation in Patients with Early-Stage Knee Osteoarthritis

**DOI:** 10.1371/journal.pone.0079785

**Published:** 2013-11-06

**Authors:** Jasper van Tiel, Max Reijman, Pieter K. Bos, Job Hermans, Gerben M. van Buul, Esther E. Bron, Stefan Klein, Jan A. N. Verhaar, Gabriel P. Krestin, Sita M. A. Bierma-Zeinstra, Harrie Weinans, Gyula Kotek, Edwin H. G. Oei

**Affiliations:** 1 Department of Orthopedic Surgery, Erasmus MC, University Medical Center, Rotterdam, The Netherlands; 2 Department of Radiology, Erasmus MC, University Medical Center, Rotterdam, The Netherlands; 3 Department of Medical Informatics, Erasmus MC, University Medical Center, Rotterdam, The Netherlands; 4 Department of General Practice, Erasmus MC, University Medical Center, Rotterdam, The Netherlands; 5 Department of Biomechanical Engineering, Delft University of Technology, Delft, The Netherlands; Faculté de médecine de Nantes, France

## Abstract

**Introduction:**

Viscosupplementation with hyaluronic acid (HA) of osteoarthritic (OA) knee joints has a well-established positive effect on clinical symptoms. This effect, however, is only temporary and the working mechanism of HA injections is not clear. It was suggested that HA might have disease modifying properties because of its beneficial effect on cartilage sulphated glycosaminoglycan (sGAG) content. Delayed gadolinium-enhanced MRI of cartilage (dGEMRIC) is a highly reproducible, non-invasive surrogate measure for sGAG content and hence composition of cartilage. The aim of this study was to assess whether improvement in cartilage structural composition is detected using dGEMRIC 14 weeks after 3 weekly injections with HA in patients with early-stage knee OA.

**Methods:**

In 20 early-stage knee OA patients (KLG I-II), 3D dGEMRIC at 3T was acquired before and 14 weeks after 3 weekly injections with HA. To evaluate patient symptoms, the knee injury and osteoarthritis outcome score (KOOS) and a numeric rating scale (NRS) for pain were recorded. To evaluate cartilage composition, six cartilage regions in the knee were analyzed on dGEMRIC. Outcomes of dGEMRIC, KOOS and NRS before and after HA were compared using paired *t*-testing. Since we performed multiple *t*-tests, we applied a Bonferroni-Holm correction to determine statistical significance for these analyses.

**Results:**

All KOOS subscales (‘pain’, ‘symptoms’, ‘daily activities’, ‘sports’ and ’quality of life’) and the NRS pain improved significantly 14 weeks after Viscosupplementation with HA. Outcomes of dGEMRIC did not change significantly after HA compared to baseline in any of the cartilage regions analyzed in the knee.

**Conclusions:**

Our results confirm previous findings reported in the literature, showing persisting improvement in symptomatic outcome measures in early-stage knee OA patients 14 weeks after Viscosupplementation. Outcomes of dGEMRIC, however, did not change after Viscosupplementation, indicating no change in cartilage structural composition as an explanation for the improvement of clinical symptoms.

## Introduction

Knee osteoarthritis (OA) is the most common joint disease in middle-aged and elderly, causing serious morbidity and large socio-economic impact [1-3]. The current treatment strategies for OA, however, are limited and end-stage OA is treated with invasive joint replacement surgery. An important drawback of this surgery is the limited durability of joint prostheses and hence the need for revision if implanted in relatively young patients. Therefore, OA research focuses on the development of disease modifying osteoarthritic drugs (DMOADs) which may allow treatment before OA reaches its end-stage [4]. Hyaluronic acid (HA) improves the viscoelastic properties of synovial fluid [5] and intra-articular injections with HA are nowadays frequently used as a viscosupplement in the treatment of knee OA [6]. Recently, a Cochrane review on the efficacy of viscosupplementation with HA in knee OA reported significantly good, but temporary clinical effects on pain, function and patient global assessment with the highest effect sizes between 5 and 14 weeks after viscosupplementation if high-molecular-weight HA derivatives are used [7]. 

The working mechanism of HA injections, however, is not yet clear. It has been suggested that viscosupplementation, in addition to symptomatic benefits, may also have disease modifying properties in OA [8,9]. As a possible pathway for disease modification, previous *in-vitro* research showed that HA has a beneficial effect on chondrocytes that are stimulated to produce proteoglycans (PG) [10-13]. PGs, which mainly consist of sulphated glycosaminoglycan (sGAG), are one of the main components of the extracellular matrix of articular cartilage [14,15]. It is known that PGs are depleted in the early stages of OA, long before cartilage degeneration is visible as joint space narrowing on radiography [16]. Therefore, radiography is considered an inappropriate imaging tool for detection and follow-up of early-stage OA in clinical research [17]. Moreover, common magnetic resonance imaging (MRI) techniques that assess cartilage morphology alterations have also shown to be insensitive to detect subtle changes in biochemical cartilage composition [18,19]. In order to diagnose OA in early-stage disease and detect intervention-caused biochemical changes sensitively during follow up, sophisticated MRI techniques have been developed during the last decade. These techniques provide a quantitative measure of the amount of sGAG, collagen or sodium of articular cartilage and therefore are a measure for cartilage structural composition [20,21]. 

An example of such a MRI technique to measure cartilage structural composition is delayed gadolinium-enhanced MRI of cartilage (dGEMRIC). The technique uses the inverse relation between a negatively charged contrast agent and the sGAG content of cartilage and therefore provides an indirect quantitative outcome measure for cartilage sGAG content [22,23]. Because of its ability to serve as a non-invasive indirect measure for cartilage structural composition, dGEMRIC has become a standard for assessment of articular cartilage sGAG content in OA research. Recently, dGEMRIC was also shown to be a highly reproducible surrogate outcome measure of cartilage sGAG content over time in early-stage OA knees [24]. Since other direct outcome measures such as cartilage biopsies are usually not ethically accepted, dGEMRIC is considered a suitable tool to non-invasively evaluate potential structure modification in terms of sGAG content improvement in articular cartilage.

Based on the aforementioned literature, we hypothesize that the improvement in clinical symptoms after HA injections will be corroborated by an improvement in sGAG content in the articular cartilage. Therefore, the aim of this study was to assess whether improvement in structural composition of cartilage is detected using dGEMRIC 14 weeks after 3 weekly injections with HA in patients with early-stage knee OA.

## Materials and Methods

### Study design and participants

For this prospective follow-up study conducted between March and September 2011, we recruited and included 20 participants with early-stage OA of the knee from the outpatient clinic of the Department of Orthopedic Surgery of our institution. This sample size was based on an expected difference in T1 relaxation time of at least 95 ms which has been shown to represent a clinically relevant improvement in cartilage sGAG content as measured by 3D dGEMRIC of early-stage OA knees acquired at 3.0 Tesla [24], a standard deviation of 100 ms of T1 relaxation times with 3D dGEMRIC of early-stage OA knees acquired at 3.0 Tesla, an α of 0.008 (corrected for multiple testing using Bonferroni-Holm method: see statistical analysis), a power of 0.8 and a maximum lost to follow-up of 10% of the included participants.

We were not able to include a control group in this study, because this was considered unethical by the Institutional Review Board (IRB). However, this was not a problem because it was not our aim to assess the potential clinical improvement of HA injections compared to a placebo or non-treated participant group. Such studies have already been performed and have shown a clinical improvement of HA injections compared to placebo [6,7]. Moreover, OA is generally a slow progressing disease in which no significant change in dGEMRIC outcomes was found at 14, 24 and 48 weeks follow-up compared to baseline in the control groups (n=15 and n=10 respectively) of two randomized controlled trials consisting of mild to moderate knee OA patients [25,26]. Thus, the absence of a control group was not considered a limitation to address our study aim, i.e. the assessment of potential sGAG *increase* in an index group treated with viscosupplementation. Based on the aforementioned results we expect that dGEMRIC would have detected minor and non-significant changes in sGAG content of the cartilage in a control group of non-treated early-stage OA patients between the baseline and follow-up measurement 14 weeks later. 

The inclusion criteria for our study were: participants age > 18 years, knee pain duration > 3 months, severity of knee pain > 2 out of 10 on a numeric rating scale (NRS) for pain (score from 0 - 10: the higher the score, the more knee pain) [27], and radiographic knee OA with a Kellgren and Lawrence grade of 1 or 2 [28]. Exclusion criteria were: viscosupplementation in the index knee within the last year, glucocorticoid injection(s) in the index knee within the last three months, absolute contra-indications to undergo MRI, renal insufficiency (glomerular filtration rate < 60 ml/min), a history of contrast medium allergy, significant co-morbidities in the lower extremity containing the index knee joint, knee surgery in the index knee within the last year or knee surgery scheduled in the index knee within the next half year.

Written informed consent was obtained from all participants and the study was approved by the IRB (Medical Ethical committee of the Erasmus MC, protocol number MEC-2010-088).

### Study protocol

Within two weeks before viscosupplementation of the index knee, a baseline dGEMRIC examination and routine MRI sequences of the index knee were acquired in all participants. Participants were also asked to rate their knee complaints on the Knee injury and Osteoarthritis Outcome Score (KOOS) [29]. The KOOS consists of 5 subscales (score from 0-100, the lower the score, the more knee symptoms in that subscale): ‘pain’, ‘symptoms’, ‘activities of daily living’ (ADL), ‘sport and function’ (sport), and ‘knee-related quality of life’ (QoL) and was validated in Dutch for early-stage OA patients by De Groot et al. in 2008 [30]. In addition to the KOOS, all participants were asked to rate their knee pain on a NRS for pain. The NRS is a numeric rating scale for pain (score from 0 - 10: the higher the score, the more knee pain) comparable with the visual analogue scale for pain, but is easier to use for patients because pain can be expressed as a number [27].

After obtaining the baseline measurements, viscosupplementation was performed using an intra-articular injection with Hylan G-F 20 (Synvisc®, Genzyme Corp, Cambridge, USA). With a time interval of one week between the injections, three injections with Hylan G-F 20 were provided by an experienced orthopedic surgeon according to a standardized protocol using a superolateral approach [31].

Follow-up measurements (dGEMRIC examination and routine MRI sequences, KOOS questionnaire, and NRS for pain) were obtained 14 weeks after viscosupplementation with HA. We chose a 14 weeks interval between viscosupplementation and follow-up measurements because the highest clinical effect sizes of intra-articular injections with HA have been reported between 5 and 14 weeks after treatment [7].

### Acquisition of dGEMRIC and routine MRI sequences

Before MR imaging, a double dose (0.2 mmol/kg) of gadopentetate dimeglumine (Magnevist®, Bayer Schering AG, Berlin, Germany) was injected intravenously based on the participants’ weight [32]. For the follow-up dGEMRIC examination, we used the same amount of contrast agent as we used for the baseline dGEMRIC examination. This way, the outcomes of the follow-up dGEMRIC are not biased by the participants’ body mass index (BMI) [33]. After contrast administration, the participants were asked to cycle for 10 minutes on a home trainer at constant speed to promote contrast distribution into and throughout the knee and the articular cartilage [34]. After cycling and a delay of 80 minutes, the dGEMRIC images were acquired.

MR imaging was performed on a 3.0 Tesla MRI scanner (Discovery MR750, General Electric Healthcare, Milwaukee, WI, USA) using a custom made 3 channel knee coil (Flick Engineering Solutions B.V., Winterswijk, The Netherlands) [24,35]. We used a three-dimensional (3D) dGEMRIC protocol which was acquired in the sagittal plane and was previously published by McKenzie et al. [36]. The dGEMRIC protocol consisted of an inversion recovery fast spoiled gradient-echo sequence, which was acquired for five times with different inversion times (TI=2100; 800; 400; 200 and 100 ms). The other scanning parameters were constant during scanning: matrix 256 x 232 pixels; field of view 150 mm; slice thickness 3 mm; flip angle 15°; echo time 1.5 ms and repetition time (TR) 3.9 ms, pixel bandwidth 488 Hz/voxel and number of averages 1. The total acquisition time was approximately 14 minutes, resulting in 36 sagittal MR images with complete coverage of the knee joint. 

In addition to dGEMRIC scans, three routine sequences consisting of a fast spin echo (FSE) proton density weighted sequence (sagittal and axial plane) and a coronal FSE T2-weighted sequence with fat suppression were acquired to allow morphological evaluation of the cartilage and incidental findings (e.g., chondroid tumors, bone tumors, etc.) in the knee. The scanning time of the additional sequences was approximately 11 minutes, resulting in a total scanning time of approximately 25 minutes for the entire MRI protocol.

### dGEMRIC analysis

Using Matlab (R2011a, The MathWorks, Natick, MA, USA), three cartilage regions of interest (ROIs) were drawn manually on three consecutive images through the lateral and medial tibiofemoral joint (central image and one adjacent image on each side) by a researcher with a medical degree and 4 years of experience in this research field (JvT). These qualifications were considered sufficient, especially since Tiderius and colleagues showed that the experience of the investigator does not affect the variability of manual ROI selection in dGEMRIC [37]. 

**Figure 1:**
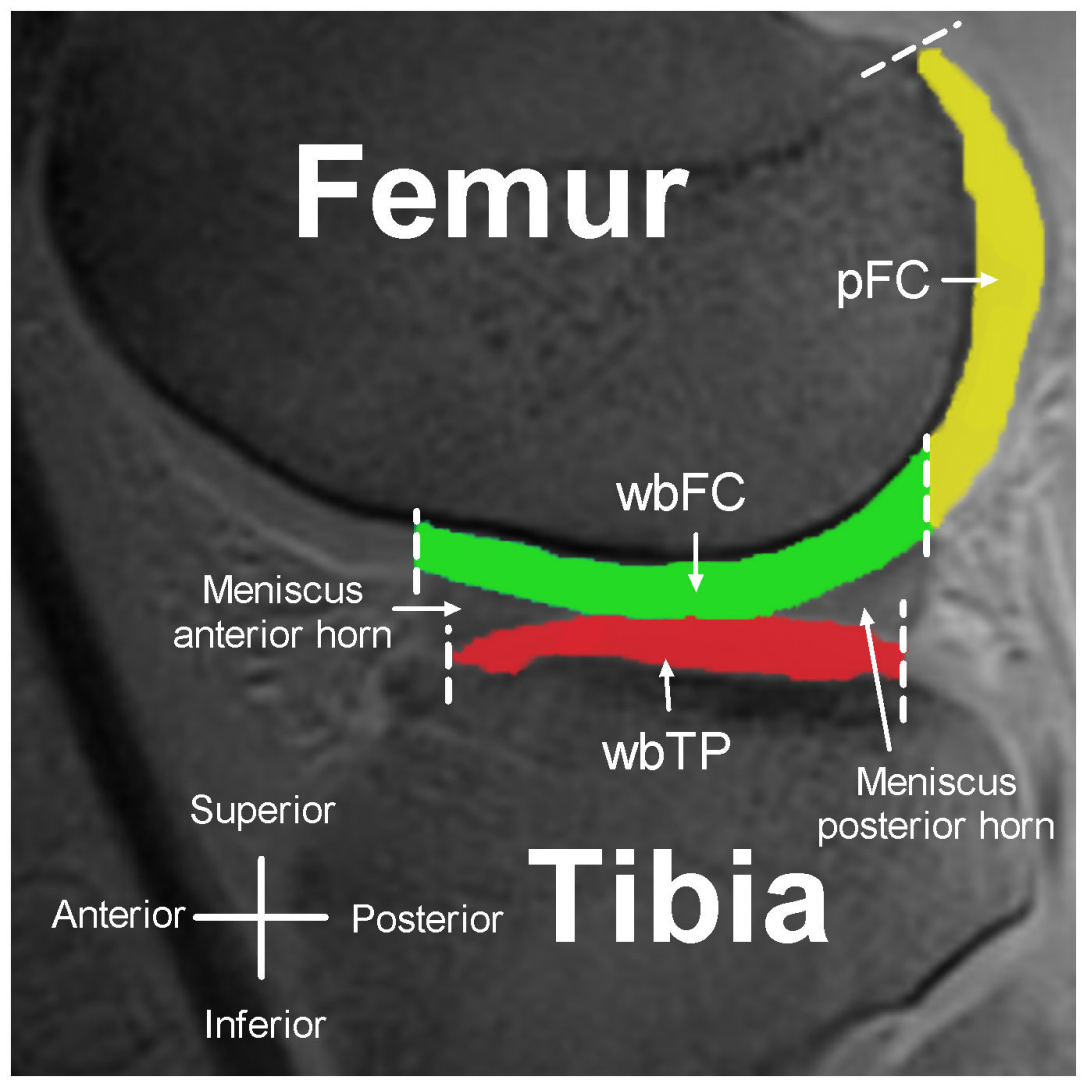
*Cartilage regions* analyzed using dGEMRIC. Central sagittal MR image through the lateral tibiofemoral joint. The three anatomical cartilage ROIs which were drawn and analyzed on three consecutive images in each compartment of the tibiofemoral joint are shown. wbFC (green): weight-bearing cartilage of the femoral condyle. pFC (yellow): posterior non weight-bearing cartilage of the femoral condyle. wbTP (red): weight-bearing cartilage of the tibial plateau.

ROI selection was standardized and based on the scheme suggested by Eckstein et al. [38]. These anatomical landmark based ROIs were drawn on the TI=2100 ms images of the first dGEMRIC examination and consisted of the weight-bearing cartilage of the femoral condyles (wbFC), the posterior non weight-bearing cartilage of the femoral condyles (pFC) and the weight-bearing cartilage of the tibial plateaus (wbTP) (Figure **1**). 

During acquisition of dGEMRIC, patient motion might occur. This patient motion may cause errors and imprecision in the outcomes of dGEMRIC, but image registration can correct for patient motion within dGEMRIC [39]. To correct for patient motion, we used an in-house developed registration and T1-fitting algorithm (Software for Post-processing And Registration of Cartilage of the Knee: SPARCK) that was previously published [35]. In the registration part of the algorithm, first all images with different TI values were aligned to the TI=2100 ms images. The femoral condyle and tibial plateau were registered separately. The images were registered using a 3D rigid transformation model by maximization of localized mutual information. To minimize the blurring of the registered images, cubic interpolation was used [40]. The registration was performed separately for the baseline and follow-up dGEMRIC acquisitions. All registrations were performed using open source registration software (Elastix, http://elastix.isi.uu.nl/) [41]. After the first step, the follow-up examination is registered to the baseline examination based on the images with TI=2100 ms, the other TI images of the follow-up acquisition are transformed accordingly. Automated registration of baseline and follow-up scans eliminates subjective visual slice matching and also eliminates the need to manually outline the cartilage ROIs in the follow-up scan. 

For the registered dGEMRIC baseline and follow-up datasets, T1 maps were estimated using a maximum likelihood fit [35]. After injection with Magnevist®, cartilage regions with long T1 relaxation time have relatively high sGAG content compared to cartilage regions with short T1 relaxation time which indicates reduced sGAG content [23,34]. All possible partial volume pixels for the cortical bone in the cartilage ROIs were automatically excluded for the ROIs using a patient specific bone-cartilage threshold which removed bone pixels for the manually drawn ROI before calculating the T1 relaxation time in all ROIs. Finally, in all cartilage ROIs, the weighted T1 relaxation time per ROI was calculated, where the estimated T1 relaxation time of each voxel was weighted by the reciprocal of its uncertainty. The uncertainty was measured by the square root of the Cramér-Rao Lower Bound, which gives a lower bound for the standard deviation of the estimated T1 [42-44]. Residual misalignment of the T1-weighted images, especially at tissue boundaries, results in biologically implausible values of T1, often associated with great uncertainty. Using the weighted mean, these implausible T1 relaxation times will not heavily influence the calculated mean T1 relaxation times in the determined cartilage ROIs [35]. 

The weighted T1 relaxation times for each anatomical cartilage ROI were averaged over the three consecutive MR images. This way we used the available 3D information instead of only using a single MR slice (2D analysis) in both the medial and lateral compartment of the knee as in most previous studies using dGEMRIC. Thus, for each patient in each dGEMRIC examination, six weighted average T1 relaxation times from six anatomical cartilage ROIs were obtained.

### Morphological cartilage analysis

On the routine MRI sequences, the articular cartilage was scored for cartilage defects according to the MRI Osteoarthritis Knee Score (MOAKS) as described by Hunter et al. [45]. Both the baseline and the follow-up MRI were read by an experienced musculoskeletal radiologist (EO).

### Statistical analysis

We tested our data for normality and equal variance using the Kolmogorov-Smirnov and Levene’s test. The outcomes of these tests showed normality and equal variance of our data. We used paired *t*-tests to compare the outcomes of dGEMRIC in each anatomically defined cartilage ROI between follow-up and baseline. The same tests were used to compare the outcomes of each KOOS subscale and the NRS for pain 14 weeks after HA injections with the baseline outcomes. Since six cartilage ROIs and six subscales of questionnaires (KOOS and NRS were analyzed together) were compared between baseline and follow-up using six paired *t*-tests, we applied a Bonferroni-Holm correction [46] to define statistically significant *p*-values for these analyses. We present both the crude, as well as the adjusted *p*-values to determine whether a particular test result is statistically significant after Holm’s adjustment of the *p*-values [47]. *P*-values of < 0.05 were considered statistically significant. All analyses were performed using SPSS 20.0 (SPSS Inc., Chicago, IL, USA).

## Results

### Participants

We included 20 participants (eight female) with early-stage OA of the knee (seven left knee joints). Their mean age at the time of inclusion was 48 ± 11 years and their mean BMI was 29 ± 5 kg/m^2^.

On radiography, 11 participants had early-stage OA in the medial tibiofemoral compartment. Two participants only had OA in the lateral tibiofemoral and 7 participants had OA in both knee compartments. No incidental findings were observed on routine MRI. 

All baseline measurements were obtained two weeks (range 8 - 16 days) before viscosupplementation of the index knee with HA. The mean time between the first and second and second and third HA injection was 7 ± 0 days for all participants. All follow-up measurements were obtained 14 weeks (range 14 - 16 weeks) after viscosupplementation. All included patients completed both the baseline and the follow-up measurements.

### dGEMRIC outcomes

At baseline, mean T1 relaxation times ranged from 461 to 491 ms in the three different cartilage regions in the medial tibiofemoral compartment. The mean T1 relaxation times in the lateral compartment were higher than those in the medial compartment and ranged from 475 to 581 ms in the different regions (*p*-value = 0.0006) (Table **1**). At 14 weeks follow-up, the mean T1 relaxation times in the medial compartment of the knee ranged from 456 to 520 ms and in the lateral compartment from 498 to 579 (*p*-value = 0.04) (Table **1**). 

**Table 1:** Mean dGEMRIC T1 relaxation times (ms) at baseline and follow-up.

**Cartilage ROI**	**Mean T1 at baseline (95% CI)**	**Mean T1 at follow-up (95% CI)**	**Crude *p*-value from paired *t*-tests**	**Adjusted p-value using Holm’s method** [46]
*Lateral tibiofemoral compartment*				
Weight-bearing femoral	512 (478 - 546) ms	510 (482 - 538) ms	0.89	0.89
Posterior femoral	475 (434 - 516) ms	487 (450 - 524) ms	0.42	> 0.99
Weight-bearing tibia	581 (529 - 633) ms	579 (526 - 630) ms	0.85	> 0.99
*Medial tibiofemoral compartment*				
Weight-bearing femoral	461 (417 - 505) ms	456 (411 - 500) ms	0.64	> 0.99
Posterior femoral	488 (432 - 544) ms	520 (470 - 569) ms	0.04	0.24
Weight-bearing tibia	491 (441 - 541) ms	512 (466 - 558) ms	0.09	0.45

Mean T1 relaxation times with 95% confidence interval for the mean in milliseconds at baseline and at 14 weeks follow-up after HA injections (*n*=20 for each anatomical cartilage ROI). 95% CI: 95% confidence interval.

**Figure 2:**
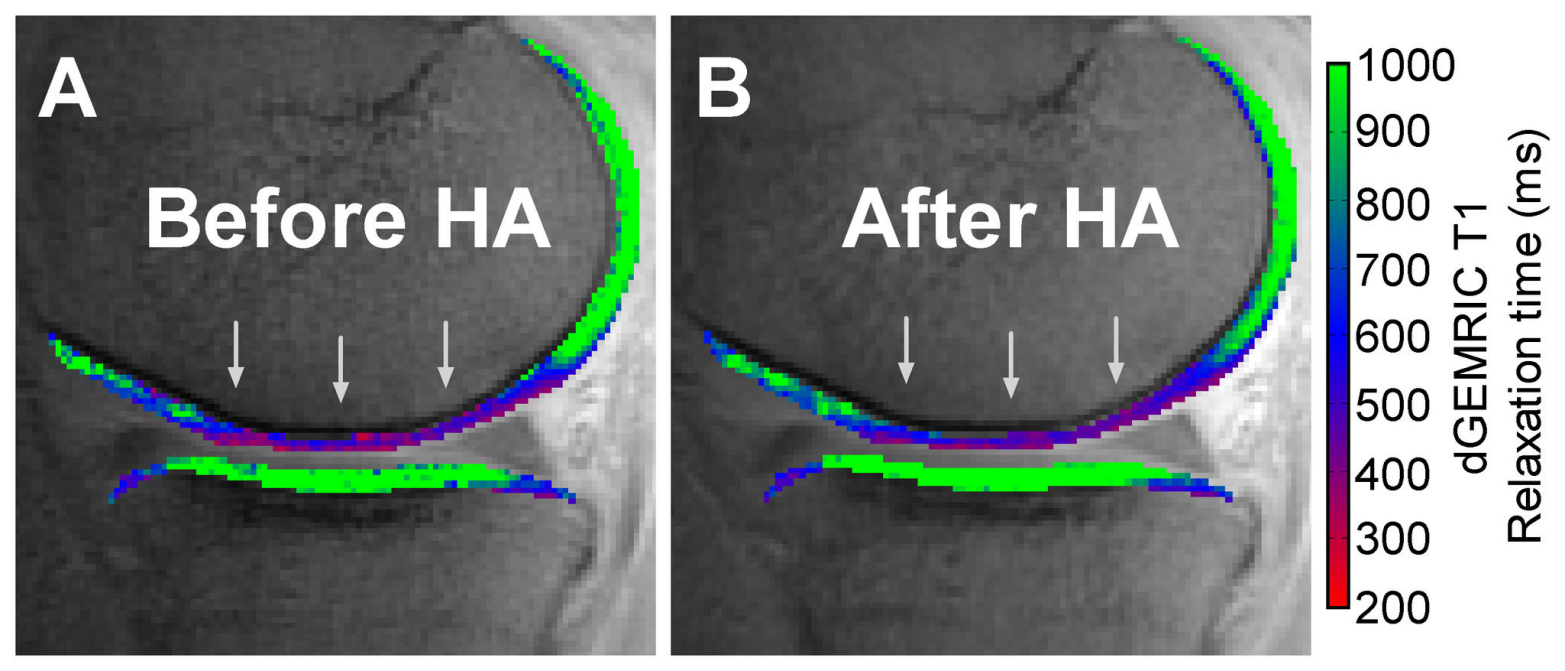
dGEMRIC color overlay representing cartilage sGAG content at baseline and follow-up. Representative sagittal central MR image through the medial tibiofemoral compartment of an early-stage OA knee before (A) and after (B) viscosupplementation with HA. The T1 color map of the cartilage clearly shows a region with relatively lower T1 relaxation times (grey arrows in A), indicating relatively lower sGAG content in the weight-bearing femoral cartilage before viscosupplementation (A). After HA injections (B), however, the region with relatively low T1 relaxation times is still present (grey arrows in B).

We did not observe a statistically significant change in T1 relaxation times in any of the analyzed cartilage regions between the baseline measurements and the follow-up measurements (all adjusted *p*-values > 0.05) (Table **1**). In Figure **2**, an example of a participant without change in cartilage T1 relaxation times and hence cartilage composition after HA injections is shown. 

**Figure 3:**
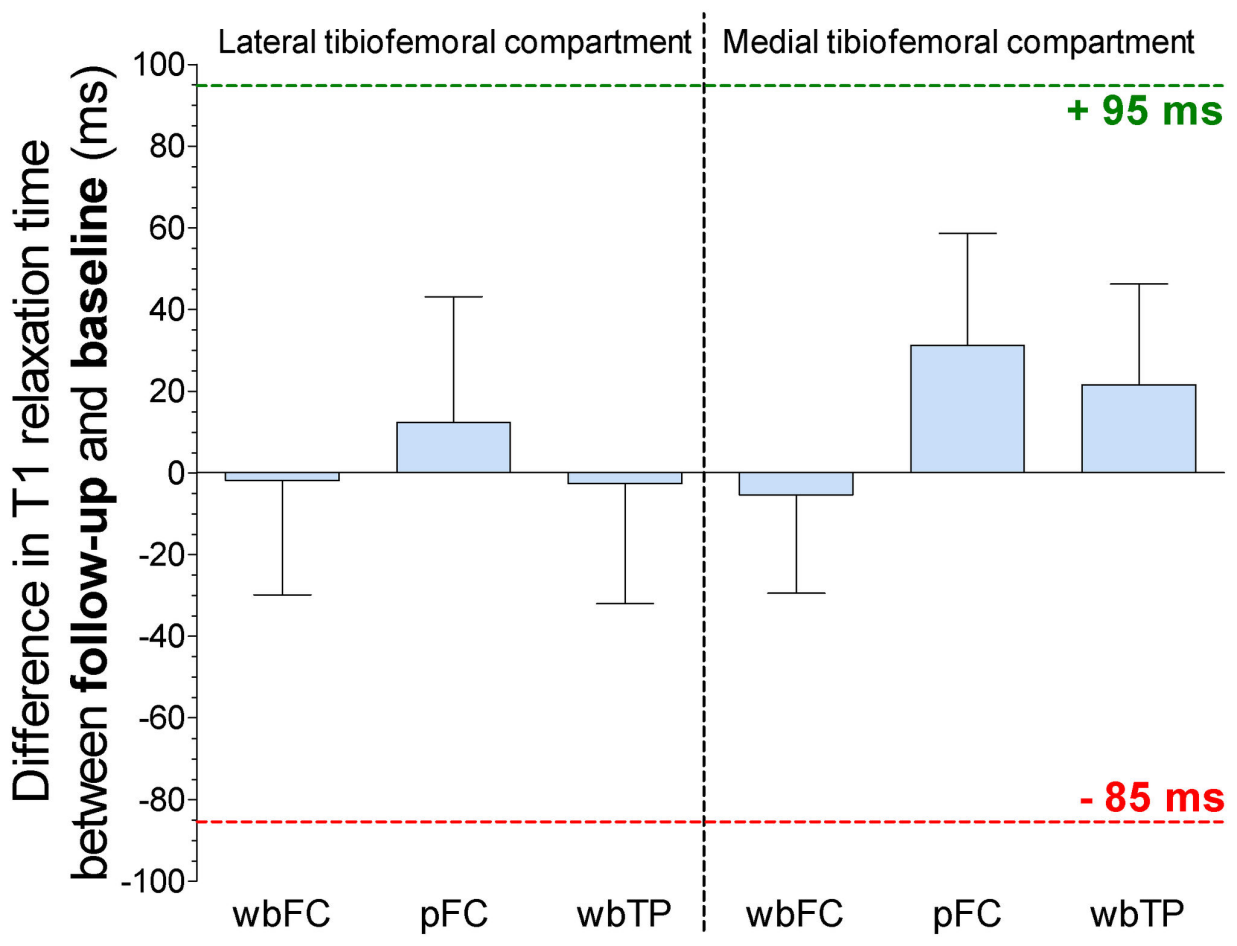
Differences between dGEMRIC T1 relaxation times at baseline and follow-up. Bar graphs showing the differences in dGEMRIC T1 relaxation times in each anatomical cartilage ROI at follow-up, 14 weeks after HA injections compared to baseline. The bar represents the mean and the whiskers represent the 95% confidence interval for the mean. +95 ms: clinically relevant threshold for improvement in cartilage sGAG content if a single patient is followed over time using dGEMRIC [24]. -85 ms: clinically relevant threshold for impairment of cartilage sGAG content if a single patient is followed over time using dGEMRIC [24]. wbFC: weight-bearing cartilage of the femoral condyle. pFC: posterior non weight-bearing cartilage of the femoral condyle. wbTP: weight-bearing cartilage of the tibial plateau.

In two ROIs (wbFC and wbTP) in the lateral knee compartment and one ROI in the medial compartment (wbFC), a trend towards a decrease in indirectly measured cartilage sGAG content in terms of lower mean T1 relaxation times was observed (Figure **3**). In one ROI in the lateral compartment (pFC) and in two ROIs in the medial compartment (pFC and wbTP), mean T1 relaxation times after viscosupplementation showed a trend towards improvement compared to baseline (Figure **3**). These trends were, however, neither statistically significant, nor exceeded a previously determined threshold of 95 ms for clinically relevant improvement in cartilage sGAG content measured using dGEMRIC [24]. 

Differences in T1 relaxation times at follow-up reached this threshold only in seven participants in a single cartilage ROI (3 times lateral pFC, 3 times medial pFC and 1 time lateral wbTP: data for each individual participant not shown). In the other ROIs in these participants, this 95 ms threshold was not reached. In the remaining 13 participants, the threshold for T1 improvement was not reached in any of the analyzed cartilage ROIs.

### Morphological cartilage analysis

On the routine MRI sequences, a total of 240 cartilage regions were assessed for cartilage lesions using the MOAKS criteria. A total of 21 regions were diagnosed with a cartilage lesion (6 in the lateral and 15 in the medial compartment) and 219 regions showed normal cartilage morphology. Eight full thickness cartilage defects were observed at baseline in 3 participants. Thirteen partial cartilage lesions were observed in 11 participants. All cartilage lesions had a size of either <10% or 10-75% of the region of cartilage surface area. No progression in any partial and full thickness size or progression from partial to full thickness cartilage lesions were observed at 14 weeks follow-up. 

### KOOS and NRS outcomes

**Figure 4:**
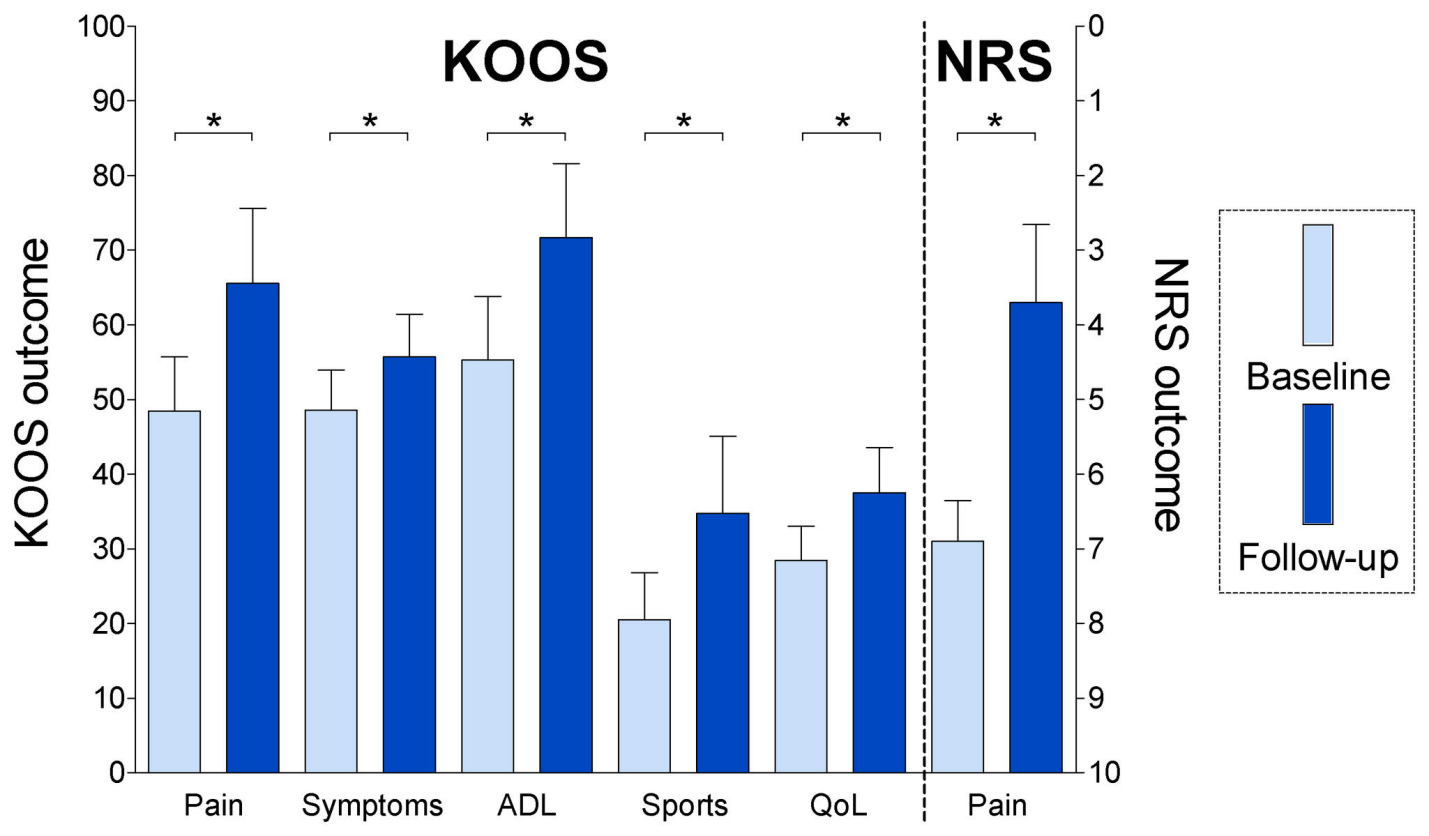
Outcomes of KOOS and NRS questionnaires at baseline and follow-up. Bar graphs showing the KOOS subscales and NRS pain at baseline (light blue box) and at follow-up, 14 weeks after HA injections (dark blue box). The bar represents the mean and the whiskers represent the 95% confidence interval for the mean. HA: hyaluronic acid injections. ADL: activities of daily living, sports: sport and function, and QoL: knee-related quality of life. *: *p*-values adjusted using Holm’s method <0.05.

All KOOS subscales, ‘pain’ (mean at baseline: 48, mean at follow-up: 66, crude *p*-value = 0.0003, adjusted *p*-value = 0.002), ‘symptoms’ (mean at baseline: 49, mean at follow-up: 56, crude *p*-value = 0.03, adjusted *p*-value = 0.03), ‘ADL’ (mean at baseline: 55, mean at follow-up: 72, crude *p*-value = 0.0007, adjusted *p*-value = 0.003), ‘sports’ (mean at baseline: 20, mean at follow-up: 35, crude *p*-value = 0.004, adjusted *p*-value = 0.01), and ‘QoL’ (mean at baseline: 28, mean at follow-up: 38, crude *p*-value = 0.01, adjusted *p*-value = 0.02), improved significantly 14 weeks after HA injections in the knee (Figure **4**). The mean NRS pain score at baseline was 7 and improved significantly (crude *p*-value < 0.0001, adjusted *p*-value < 0.0001) to a mean of 4 14 weeks after HA injections (Figure **4**). 

## Discussion

Because of the lack of established DMOADs for early-stage knee OA, intra-articular viscosupplementation with HA has become a frequently used treatment for reducing symptoms and pain in early-stage knee OA [6,7]. Since it was suggested in previous *in-vitro* research that HA injections might also have disease modifying properties by increasing cartilage sGAG content [10-13], the aim of this study was to assess whether improvement in cartilage structural composition is detected using dGEMRIC 14 weeks after 3 weekly injections with HA in patients with early-stage knee OA.

Outcomes of dGEMRIC in the medial compartment of the knee were lower compared to the lateral compartment at baseline, indicating more structural damage in the medial knee compartment. T1 relaxation times in both knee compartments were lower compared to previously published dGEMRIC T1 relaxation times acquired at 3.0 Tesla in healthy subjects [36,48,49], indicating sGAG loss from the cartilage in our early-stage OA patients. The outcomes of dGEMRIC are consistent with radiographic findings and our morphological cartilage assessment on MRI with MOAKS and reflect the early-stage OA population in which sGAG loss occurs before morphological changes are detectable on radiography or MRI (e.g. using MOAKS). We observed early-stage OA in the medial compartment of the knee in 18 of the 20 participants, which is defined as mild to moderate osteophyte formation as the only features on radiography, and only a few participants with partial cartilage damage according to MOAKS, without definite joint space narrowing or bone on bone contact which are signs of advanced or end-stage OA. Based on these characteristics, we consider our study population suitable to evaluate the potential structural effects of viscosupplementation as a potential DMOAD, since this should be tested in early stages of OA in which disease modification is still possible [4]. 

At follow-up, 14 weeks after viscosupplementation with HA, no statistically significant change in cartilage sGAG content was detected on dGEMRIC in any of the analyzed anatomical cartilage ROIs compared to the baseline measurements. Outcomes of dGEMRIC showed a trend towards improvement in three of the analyzed cartilage ROIs two of which were the medial and lateral non-weight-bearing cartilage regions of the femoral condyles. This is somewhat unexpected, as one would expect an improvement in cartilage structural composition in the weight-bearing femoral condyles and/or plateaus since those ROIs had lower T1 relaxation times and hence more structural damage at baseline. Moreover, the improvement in T1 relaxation times did not exceed a previously determined threshold of 95 ms which has been shown to represent a clinically relevant improvement in cartilage sGAG content as measured by 3D dGEMRIC of early-stage OA knees acquired at 3.0 Tesla [24]. It may be that there are non-significant changes between baseline and follow-up T1 relaxation times which means that the measurements were not the same between the two time points. However, we hypothesized that if the reported clinical effect of viscosupplementation, confirmed by our study, would act through an *improvement* of sGAG content, this would have been detectable using dGEMRIC with the sample size of our study.

We believe there are several possible explanations why we did not observe an increase in cartilage sGAG content by dGEMRIC 14 weeks after viscosupplementation of early-stage OA knees. First, cartilage sGAG content and therefore dGEMRIC outcomes might not improve after viscosupplementation if the treatment does not have any disease modifying effects on articular cartilage. Instead, viscosupplementation may have a primary positive effect on pain and other clinical symptoms of OA. This working mechanism was suggested for HA injections in a recent OARSI review by Zhang et al. in which the evidence for available therapies in the treatment of hip and knee OA was re-evaluated and discussed [50]. 

A second explanation why T1 relaxation times did not increase is that viscosupplementation may *slow down the progression* of OA by preventing the loss of sGAG content rather than *improving* the sGAG content of cartilage (the latter was our hypothesis in this study). This working mechanism of HA was suggested in several *in vitro* studies [51-53], and is supported by the results of recent animal and human studies in which the structural efficacy of HA treatment over time was compared to a control group (non-treatment and placebo) using another quantitative MRI technique (T2 mapping) [54] and cartilage thickness and volume measurements on MRI [55]. However, as it was considered unethical to include a control group by the IRB, we could not compare the sGAG content 14 weeks after viscosupplementation with the sGAG content of cartilage without viscosupplementation.

A third possible reason that T1 relaxation times may not increase after HA injections is the detection limit and specificity of dGEMRIC to detect (change) in sGAG content of articular cartilage. Minimal changes in sGAG content of cartilage following HA treatment may not be detected using dGEMRIC T1 because, although the technique is highly reproducible in knee OA [24], it is currently unknown to which extent minimal change in sGAG content are detectable using dGEMRIC in humans. dGEMRIC is an indirect measure for cartilage sGAG content and there are no *in vivo* studies which investigated the sensitivity and specificity of dGEMRIC to measure (small) changes in sGAG content of the extracellular matrix of cartilage. Other drawbacks of dGEMRIC are the long acquisition protocol because of the delay between the contrast administration and MR acquisition and the risk of nephrogenic systemic fibrosis due to contrast administration. In addition to these drawbacks, a recent publication shows that dGEMRIC outcomes might not only represent sGAG content of cartilage, but may also be influenced by collagen orientation which influences diffusion of contrast agent into the extracellular matrix of the cartilage [56] and therefore concluded that dGEMRIC may not be considered sGAG specific. Despite these shortcomings of dGEMRIC, the technique is still considered the best tool available that provides a quantitative measure for cartilage sGAG content in human knee joints. 

Finally, the timing of the follow-up measurement in our study could be an explanation why dGEMRIC T1 relaxation times did not improve after viscosupplementation. It may be that the follow-up measurement was either too early or too late after viscosupplementation to detect any changes in cartilage sGAG content caused by the treatment. We chose a 14 weeks follow-up period after viscosupplementation based on a Cochrane review on the efficacy of HA injections as treatment in knee OA [7], in which the maximum clinical benefit of HA injections was reached between 5 and 14 weeks after treatment. It is known that sGAGs are being synthesized within days instead of weeks [57]. Moreover, previous *in vivo* animal research has shown that newly synthesized sGAGs have a turnover time over 100 days [58,59] and therefore should be still detectable 14 weeks after viscosupplementation. Therefore, we considered our follow-up period of 14 weeks appropriate in relation to our hypothesis. Future research with earlier or extended follow-up measurements might give better insight whether our findings are consistent over time. 

In contrast to the results of dGEMRIC, all KOOS subscales (‘pain’, ‘symptoms’, ‘daily activities’, ‘sports’ and ’quality of life’) and the NRS for pain improved significantly 14 weeks after viscosupplementation with HA. These results are in agreement with aforementioned studies [6,7] in which a significant reduction in patient complaints was observed after HA injections. The relief in patient complaints without an improvement in cartilage sGAG content might be due to the placebo effect of viscosupplementation [50]. However, the clinical efficacy of HA may also be attributed to a positive effect of viscosupplementation on the viscoelastic properties of the synovial fluid [5] and a positive effect on the synovial membrane, which has been observed histologically in previous clinical studies in OA patients [60-62]. It has been suggested in previous work that this might have anti-inflammatory effects causing less synovitis and therefore less knee complaints since pain and synovitis were recently shown to be closely related in OA patients [63-65]. 

In conclusion, the results of this study confirm earlier findings reported in the literature, showing a persisting efficacy of viscosupplementation on symptomatic outcome measures of early-stage OA knees 14 weeks after treatment. Outcomes of dGEMRIC, however, did not change after viscosupplementation, indicating no change in cartilage structural composition as an explanation for the improvement of clinical symptoms.
